# Fermentative valorisation of agri-food residues: optimisation of *Lactiplantibacillus paraplantarum* fermentation conditions for the production of platform organic acids

**DOI:** 10.3389/fbioe.2026.1935066

**Published:** 2026-09-17

**Authors:** Bruno Navajas-Preciado, Sara Martillanes, Jonathan Delgado-Adámez

**Affiliations:** 1 Centro de Investigaciones Científicas y Tecnológicas de Extremadura (CICYTEX), Instituto Tecnológico Agroalimentario de Extremadura, Badajoz, Spain; 2 Centro de Investigaciones Científicas y Tecnológicas de Extremadura (CICYTEX), Incubadora de Alta Tecnología en Bioeconomía y Economía Circular, Mérida, Spain

**Keywords:** bioprocess optimisation, cascade biorefinery, heterolactic fermentation, organic acids, quantitative 1H NMR, second-generation biomass, spent coffee grounds, tomato pomace

## Abstract

**Introduction:**

Organic acid fermentation is a key route for integrating second-generation (2G) biomass into biorefineries and promoting industrial symbiosis between agri-food residues and platform-chemical production. However, plant matrices such as tomato pomace (TP) and spent coffee grounds (SCG) show limited carbon accessibility and contain phenolic and lipidic compounds that may inhibit bacterial growth.

**Methods:**

This study uses hydroalcoholically pretreated TP and develops an iterative strategy for organic acid production using Lactiplantibacillus paraplantarum as a biofactory, while evaluating selectivity towards lactate. Quantitative proton nuclear magnetic resonance was used to monitor lactic acid (LA) and acetic acid (AA) titres and apparent glucose-equivalent conversion (aGEC) into these acids. The aGEC is a comparative, non-stoichiometric index rather than a closed carbon yield, because it includes buffer-derived acetate.

**Results and Discussion:**

Response surface methodology identified 44.7% TP replacement, 79.6 h and an initial pH of 5.77 as the basal fermentation conditions (4.73 ± 0.26 gL-1 LA; aGEC ≈ 6%). Nitrogen-source optimisation and pH buffering increased LA to 7.68 ± 0.56 and 14.52 ± 0.76 gL-1 and aGEC to ≈ 10% and ≈ 26%, respectively. The final combined intensification treatment comprised cellulase pretreatment, a modified incubation atmosphere and CaCO_3_ as an additional buffering system. It increased aGEC to ≈ 85% but did not significantly increase LA. Acetate accumulated substantially, and the simultaneous modifications prevented attribution of the response to any individual factor. Transfer of the basal conditions optimised for TP to SCG was feasible only at moderate substitution levels. At 44-60% replacement of standard glucose, SCG maintained comparable aGEC values of approximately 28-29% and produced 16.92-15.87 gL-1 LA, whereas higher substitution reduced both aGEC and LA production. This sequential framework distinguishes total-acid accumulation from LA selectivity and shows the limited transferability of residue-specific fermentation conditions.

## 1 Introduction

Given the limited availability of exploitable natural resources and the need to decarbonise value chains, many traditional sectors are seeking new ways to obtain platform chemicals, including organic acids, alcohols and polymer precursors, from renewable biogenic resources rather than fossil feedstocks (European Commission, 2025). The limited availability of exploitable natural resources and the need to decarbonise value chains have driven interest in first- and second-generation (1G and 2G) biomass as a means of replacing fossil resources and strengthening industrial resilience. In particular, high-volume 2G biomass streams could support multiproduct biorefineries and circular valorisation (Prado-Acebo et al., 2024). However, their heterogeneity and recalcitrance require combinations of pretreatment and bioprocessing that complicate microbial conversion (Yankov, 2022; Pandey et al., 2025).

Organic acids such as acetic and lactic acids are already produced mainly through fermentation, but two constraints remain especially relevant: (i) the cost of refined carbon feedstocks and culture-medium components, which often represents 40%–70% of the total fermentation cost (Ojo and de Smidt, 2023; Kacaribu and Darwin, 2024), and (ii) the current reliance on 1G biomass as the main fermentable substrate, including maize starch and sugarcane juice (de França et al., 2022). This reliance competes directly with food uses and therefore strengthens the need to prioritise food supply over industrial products, particularly in view of the expected growth in global demand (FAO et al., 2023). For this reason, 2G biomass, despite its more complex management, has gained interest because it does not directly compete with food supply. In this context, TP and SCG are attractive because both arise from large agri-food chains and can be standardised after primary processing. Tomato processing generates an estimated 0.6–2.0 million tonnes of tomato pomace (TP) worldwide each year (Chabi et al., 2024). For spent coffee grounds (SCG), the five largest coffee-producing countries produced approximately 7.0 million tonnes of green beans in 2022. Applying the reported generation factor of 0.60–0.65 tonnes SCG per tonne of green coffee gives a conservative lower-bound estimate of approximately 4.2–4.6 million tonnes of SCG annually (Wysocki et al., 2024).

Current biorefineries based on 1G biomass and extensively pretreated lignocellulosic biomass can achieve LA titres above 100 g L^-1^ (Ren et al., 2022; Zhang et al., 2023), setting a demanding benchmark for residual 2G substrates. TP and SCG remain relatively underexplored fermentation feedstocks, and a clearly defined operating range has not yet been established for broths containing a high proportion of solid agri-food residues (Wysocki et al., 2024; Paniagua-García et al., 2024).

For TP, the literature describes a matrix dominated by structural fractions, mainly skins and seeds, with a high proportion of fibre. Cellulose and hemicellulose contents of 39%–59% on a dry basis have been reported (Becze et al., 2025), while tomato-skin characterisations indicate approximately 53% total lignin (Benito-Román et al., 2025). These values anticipate high recalcitrance and limited accessibility to fermentable carbohydrates without prior treatment. Accordingly, organic-acid fermentation of TP commonly involves chemical hydrolysis to remove non-cellulosic components and obtain glucose-enriched fractions (Pirozzi et al., 2022), or enzymatic treatment with pectinase and cellulase to release mono- and oligosaccharides (Scaglia et al., 2023). TP also concentrates polyphenols and carotenoids that may restrict microbial performance (López-Téllez et al., 2024; Plaza et al., 2023; Hatami and Ciftci, 2024). SCG presents analogous constraints, including lignocellulosic recalcitrance and phenolic and lipid fractions that can inhibit microorganisms (Wysocki et al., 2024; Kim S. et al., 2024; Abolore et al., 2024).

Evidence from mixed-culture acidogenic fermentation further shows that acid yields from food-industry by-products are strongly influenced by both matrix characteristics and fermentation strategy. In this context, sonication has been shown to improve the performance of a recalcitrant grape-pomace stream, further highlighting the importance of substrate accessibility (Marchetti et al., 2023).

These compositional and process-related constraints indicate that the challenge extends beyond improving substrate accessibility; it also requires microbial platforms capable of tolerating residual inhibitory compounds and efficiently converting the resulting complex sugar mixtures into target products. Lactic acid bacteria (LAB), particularly heterofermentative strains commonly associated with plant-derived residues (Yang et al., 2010), exhibit substantial potential as microbial cell factories for converting these substrates into organic acids (Mora-Villalobos et al., 2020). Nevertheless, the metabolic versatility that allows LAB to utilise heterogeneous plant matrices may pose a challenge when high selectivity for a specific acid is desired. Factors such as stress responses, redox balance, and substrate composition can redirect a portion of the carbon flux away from lactate, leading to mixed-acid fermentation profiles (Tejedor-Sanz et al., 2022; Wysocki et al., 2024).

While TP fermentation has been investigated, it remains uncertain whether sequential optimisation can effectively differentiate nutritional, pH-related, and substrate-accessibility constraints in a high-solids TP medium. Additionally, it is unclear whether the resulting basal conditions are applicable to another 2G matrix.

Based on these gaps and building on previous work focused on the green extraction of bioactive compounds from TP (Navajas Preciado et al., 2026b), this study continues a cascade biorefinery approach by using the post-extraction solid residue of TP as a fermentation feedstock. Specifically, the aim was to optimise the fermentation parameters of the heterofermentative bacterium *Lactiplantibacillus paraplantarum* for the valorisation of pretreated TP into platform organic acids and to assess whether the resulting optimised basal fermentation parameters could be applied to SCG. The optimisation was conducted sequentially, first defining the basal growth conditions by response surface methodology (RSM), and then refining nitrogen supplementation and pH buffering according to the physiological constraints of the strain. Cellulase pretreatment and atmosphere control were subsequently evaluated as external intensification variables, while similarly pretreated SCG was used to determine whether the optimised TP conditions could be transferred to another complex 2G biomass without redesigning the process from the beginning. By separating basal optimisation from process intensification, the study seeks to distinguish total-acid accumulation, LA selectivity, and cross-residue transferability.

## 2 Materials and methods

### 2.1 Raw materials and pretreatment

All chemicals and solvents were of analytical or high-performance liquid chromatography grade. Ethanol, sodium hydroxide (NaOH), hydrochloric acid (HCl), sodium chloride (NaCl), acetate buffer, calcium carbonate (CaCO_3_), deuterium oxide (D_2_O) and cellulase from *Aspergillus niger* (nominal activity ≈0.8 U mg^-1^; lot-specific activity, 1.21 U mg^-1^) were purchased from Sigma-Aldrich (St Louis, MO, United States). De Man, Rogosa and Sharpe (MRS) broth, M9 salts, glucose (Glc.), peptone, yeast extract and ammonium sulphate ((NH_4_)_2_SO_4_) were obtained from Scharlab (Barcelona, Spain). Dimethyl sulfoxide (DMSO), lactic acid (LA) and acetic acid (AA), used as standards for quantitative proton nuclear magnetic resonance (q-^1^H NMR), were supplied by Thermo Fisher Scientific (Waltham, MA, United States).

The raw materials were TP (*Solanum lycopersicum* L.) and SCG. The TP comprised peels and seeds collected after the industrial production of tomato concentrate and powder in Villanueva de la Serena, Spain, in late July 2023. The SCG were collected from households and coffee shops in Berlin, Germany. Both residues were dried for 48 h at 45 °C in a forced-air oven (Universal Oven UN110plus, Memmert, Germany; 70% airflow). The TP was pretreated using the optimised batch solid-liquid extraction protocol of Navajas Preciado et al. (2026b). The SCG were pretreated using an adaptation of the batch ethanol extraction reported for rice bran oil by Martillanes et al. (2018). The extractions used 100% ethanol at 35 °C for 133 min for TP and at 65 °C for 130 min for SCG. In both cases, the solids were recovered by filtration, washed with purified water and dried for 48 h at 45 °C with 70% airflow. These pretreatments reduced the phenolic load and, consequently, the antimicrobial pressure during fermentation.

The workflow employed in this study is illustrated in Figure 1.

**FIGURE 1:**
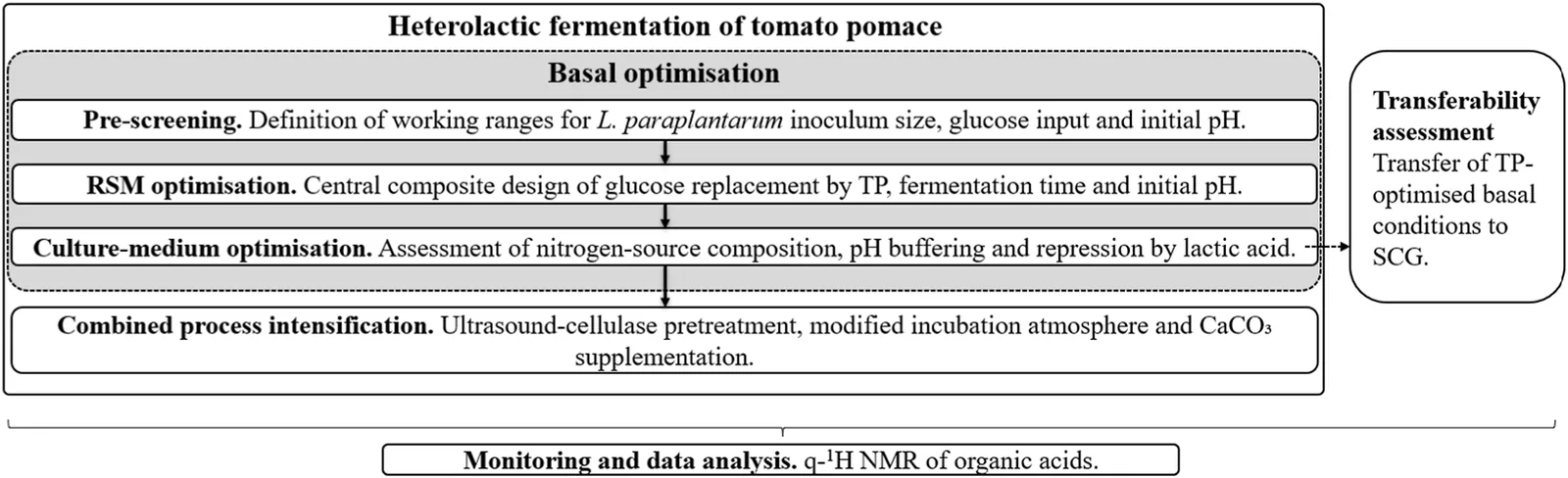
Sequential experimental strategy for optimising the heterolactic fermentation of tomato pomace by *Lactiplantibacillus paraplantarum.* TP, tomato pomace; SCG, spent coffee grounds; RSM, response surface methodology; q-^1^H NMR, quantitative proton nuclear magnetic resonance.

### 2.2 Sequential optimisation of organic acids production by *Lactiplantibacillus paraplantarum*

#### 2.2.1 Pre-screening

Pre-screening defined the factor bounds for the subsequent central composite design (CCD). A 24-well assay evaluated glucose input (0.1 or 0.5 mL of a 20% glucose stock in a final volume of 1.5 mL), inoculum size (1%–10%, vv^−1^) and initial pH (5.3–6.8; Supplementary Table S1). Before inoculation, the initial pH was adjusted with 1 M NaOH or HCl. No external buffer was added, and the pH was not subsequently adjusted. Cultures were incubated aerobically at 30 °C and 140 rpm. Colony-forming units (CFU mL^-1^) were determined on days 0 and 7. Medium pH was measured immediately after inoculation (day 0) and on days 3 and 7. Conditions were selected when the log_10_-transformed net increase in viable counts, log_10_(CFU_t7_ - CFU_t0_), was ≥7.4 and the final pH was ≤5.0.

#### 2.2.2 RSM optimisation

Fermentation parameters were optimised by combining RSM with a CCD. The independent variables were the proportion of Glc. replaced by TP (%, factor A), total fermentation time (h, factor B) and initial pH (factor C). The bounds for factors B and C were based directly on the pre-screening results. The bounds for factor A were informed by two considerations. First, dry TP contains approximately 30.6 g of total carbohydrates per 100 g, including 16.05 g of glucan (cellulose and starch). Hydrothermal pretreatment followed by enzymatic hydrolysis can achieve approximately 61.0%–66.8% saccharification, supporting the use of TP as a glucose-equivalent (Glc. eq.) feedstock (Hijosa-Valsero et al., 2019). Second, when TP or SCG formed part of the principal carbon source, Glc. eq. was calculated from glucan using the standard anhydro correction for polymer-to-monomer conversion (180/162 = 1.11), in accordance with established biomass carbohydrate accounting (Sluiter et al., 2012). These values would support a higher loading of approximately 3.5 g TP per gram of Glc. on a sugar-equivalent basis. However, the geometric constraints of the test vessel limited the loading to 1.5 g TP per gram of Glc. This loading kept the matrix fully hydrated and left one-third of the vessel as headspace for gas exchange. The resulting theoretical initial input was up to 81.9 g L^-1^ Glc. eq. and served as the reference value in all TP and SCG fermentation experiments. Supplementary Table S2 summarises the Glc. eq. ratios (X % Glc. + Y % TP) evaluated by RSM. All experiments were conducted in triplicate.

Temperature (30 °C), agitation (140 rpm), M9 salt composition and the parameters established during pre-screening were kept constant. LA production (gL^-1^) was the dependent variable and was quantified by ^1^H NMR. AA was also quantified by ^1^H NMR to calculate aGEC from the summed concentrations of LA and AA. All spectra are available in Zenodo (Navajas Preciado et al., 2026a).

##### 2.2.2.1 Sample and inoculum preparation

Autoclavable 50 mL polypropylene Falcon conical tubes were loaded with the quantity of TP specified in Supplementary Table S2. M9 solution was prepared separately, and its pH was adjusted according to the experimental design with 1 M NaOH or HCl. The TP and M9 preparations were autoclaved at 121 °C for 20 min. Glc. was dissolved in pH-adjusted M9 solution and sterilised separately by filtration through a 0.45 µm membrane. The sterile Glc. solution was then added to the tubes containing TP and M9 medium.

To prepare the inoculum, a colony of *Lactiplantibacillus paraplantarum* (CECT 5783, Colección Española de Cultivos Tipo, Valencia, Spain) from a pre-grown plate was suspended in 50 mL of MRS broth. The culture was incubated for 24 h at 30 °C with orbital shaking at 150 rpm. A 10 mL aliquot was then centrifuged at 14,000 rpm for 5 min at 15 °C, and the pellet was washed twice with 10 mL of 0.9% NaCl solution. The final pellet was resuspended in pH-adjusted M9 medium to an optical density at 600 nm (OD_600_) of 0.25–0.30.

All experimental units received a 5% (vv^−1^) inoculum (approximately 1.5 mL) and were incubated for the periods specified in Supplementary Table S2 with orbital shaking at 140 rpm.

#### 2.2.3 Nitrogen-source and pH optimisation

##### 2.2.3.1 Nitrogen-source optimisation

Samples were prepared as described in Section 2.2.2. The operational nitrogen-source:carbon-source mass ratio was calculated by dividing the total mass of peptone, yeast extract and/or (NH_4_)_2_SO_4_ by the combined mass of TP and glucose. Supplementary Table S3 gives the final composition of each experimental unit. The primary selection criterion was LA titre; aGEC and the LA:AA distribution were secondary criteria. Only the amount and composition of the nitrogen source were varied; the conditions identified by RSM were held constant.

##### 2.2.3.2 pH buffering and lactic-acid supplementation

Samples were prepared as described in Section 2.2.2 using the formulation selected in Section 2.2.3.1. Initial pH and exogenous LA were varied as shown in Supplementary Table S4. Exogenous LA was added to test whether accumulated LA suppressed further endogenous production; the concentration range was based on the nitrogen-optimisation results. The acetate buffer was adjusted either to the RSM optimum of pH 5.79 or to pH 4.5, which is above the reported lower growth limit of pH 3.8 for *L. paraplantarum*. LA and AA were quantified by q-^1^H NMR.

#### 2.2.4 Combined process intensification

The conditions selected in Section 2.2.3.2 were used to evaluate a final combined intensification treatment comprising ultrasound-cellulase pretreatment, modification of the incubation atmosphere, and supplementary buffering with CaCO_3_. TP suspended in M9 medium adjusted to pH 7 was sonicated at 360 W for 20 min. Each sample then received 0.02 g of cellulase and was incubated at 50 °C for 24 h. Samples were subsequently prepared according to Supplementary Table S5. Because higher acid titres were anticipated, every intensification treatment also received 2.3% CaCO_3_ (wv^−1^). CaCO_3_ was introduced at the same time as the other modifications, and the design did not include a basal condition supplemented only with CaCO_3_. Therefore, any increase relative to the basal condition cannot be attributed to a single factor. LA and AA were quantified by q-^1^H NMR.

#### 2.2.5 Transferability from TP to SCG

The basal conditions selected in Section 2.2.3.2 were transferred from TP to SCG without further optimisation. Given the heterogeneity of SCG, several levels of glucose replacement were evaluated (Supplementary Table S6). Samples were prepared as described in Sections 2.2.2.1-2.2.3.2. After fermentation, LA and AA were quantified by q-^1^H NMR.

### 2.3 EXPERIMENTAL-DESIGN limitations

All experiments were conducted in 50 mL polypropylene conical tubes. Continuous pH-stat control by automated NaOH addition was therefore not technically feasible without compromising sterility. Instead, pH management was evaluated sequentially under three conditions: no external buffer through the nitrogen-source optimisation stage (Supplementary Tables S2, S3); acetate buffer during the pH and lactate assessment (Supplementary Table S4) and the transferability assessment (Supplementary Table S6); and acetate buffer with CaCO_3_ during the final intensification stage (Supplementary Table S5). The acetate introduced with the buffer was not subtracted from the final AA concentration. Consequently, the conversion of Glc. eq. into LA + AA must be interpreted as an aGEC rather than a closed carbon balance. This limitation may overestimate the absolute values in buffered treatments. Values above 100% arise from the limitations of the index, which neither subtracts buffer-derived acetate nor includes other potentially fermentable carbon inputs, such as peptone and yeast extract, in the theoretical denominator. Such values must not be interpreted as physically closed conversion yields. Relative trends were therefore compared only between stages that shared the same acetate-buffer formulation, and absolute values were not treated as closed yields.

This laboratory-scale study did not include a techno-economic assessment of ethanol recovery, drying, organic nitrogen supplementation, neutralisation or downstream acid recovery. The hydroalcoholic treatment should therefore be interpreted as the first step in a potential cascade valorisation scheme for TP and SCG.

### 2.4 Analytical procedures

#### 2.4.1 q-^1^H NMR instrumentation and acquisition

^1^H NMR spectra were recorded at 298 K on two spectrometers (Bruker BioSpin, Karlsruhe, Germany): a Bruker Avance III 500 MHz instrument for the combined intensification treatment and a Bruker AvanceNeo 700 MHz instrument equipped with a Prodigy cryoprobe for the basal optimisation and transferability assessments. On the 500 MHz instrument, spectra were acquired with the zg30 pulse sequence, 128 scans and an acquisition time (aq) of 3.0 s. On the 700 MHz instrument, q-^1^H NMR spectra were acquired with the noesyggpr1d pulse sequence to suppress residual water effectively. These quantitative experiments used 64 transients and a relaxation delay (d1) of 10 s to ensure full longitudinal relaxation. Two-dimensional ^1^H/^1^H correlation spectroscopy experiments were also conducted to confirm peak assignments.

#### 2.4.2 Sample preparation

Culture broth samples were analysed either directly or after clarification by centrifugation at 11,180 × g for 5 min in a Micro Star 21R centrifuge (VWR International, Leuven, Belgium) fitted with a 24-place fixed-angle rotor for microtubes (Thermo Scientific, catalogue no. 75003424; maximum radius, 8.6 cm). Samples were prepared in 5 mm NMR tubes by combining 390 µL of culture broth, 100 µL of D_2_O for field-frequency locking and 10 µL of a 10% (vv^−1^) DMSO internal-standard stock solution. The stock solution was prepared by diluting one volume of DMSO with distilled water to a final volume of ten volumes. This preparation produced a final sample volume of 500 µL and a DMSO concentration of 2.2 g L^-1^ in each NMR sample.

#### 2.4.3 Quantification and calibration

DMSO (2.2 g L^-1^) served as the internal standard and produced a well-resolved singlet at 2.7 ppm. This signal did not overlap with the resonances of lactic acid/lactate, acetic acid/acetate or other medium components. Organic acids were quantified by manually integrating their specific ^1^H resonances relative to the internal DMSO signal.

### 2.5 Calculations and statistical analysis

#### 2.5.1 Sequential optimisation

Apparent glucose-equivalent conversion was calculated as aGEC (%) = 100 × ([LA] + [AA])/81.88 g L^-1^, where [LA] and [AA] are the measured acid concentrations and 81.88 g L^-1^ is the theoretical glucose-equivalent concentration in the 30 mL system. This mass-based, non-stoichiometric index neither subtracts buffer-derived acetate nor corrects for carbon stoichiometry or other carbon inputs and outputs. It was therefore used only for comparative interpretation, principally between treatments with the same buffer formulation.

Every experimental point was tested in triplicate, and results are presented as the mean ± standard deviation (SD). The CCD was evaluated by analysis of variance (ANOVA) to assess model adequacy, parameter significance and the main and interaction effects on the response. The data were fitted to the second-order polynomial in Equation 1, where LA is the predicted response; β_0_ is the intercept; β_1_, β_2_ and β_3_ are the linear coefficients; β_12_, β_13_ and β_23_ are the interaction coefficients; and β_11_, β_22_ and β_33_ are the quadratic coefficients for factors A, B and C. Calculations were performed in Design-Expert software (version 11.1.2.0; Stat-Ease Inc., Minneapolis, MN, United States).

$$\text{LA}=\beta_{0}+\beta_{1}A+\beta_{2}B+\beta_{3}C+\beta_{12}\text{AB}+\beta_{13}\text{AC}+\beta_{23}\text{BC}+\beta_{11}A^{2}+\beta_{22}B^{2}+\beta_{33}C^{2}$$ (1)

#### 2.5.2 Acid quantification

For all acid determinations, results are presented as the mean ± SD. After normality had been verified, differences among groups were assessed by ANOVA followed by Tukey’s honestly significant difference *post hoc* test (p < 0.05). Different letters indicate significant differences among treatments. These analyses were performed in OriginPro 2023 (version 10.0; OriginLab Corporation, Northampton, MA, United States).

Operational repeatability was evaluated from the distribution of DMSO integrals across experimental groups in a scatter-whisker plot. This analysis assessed the consistency of sample preparation and integration; it did not assess analytical accuracy or constitute full method validation. The analysis was performed in JASP 0.95.4 (University of Amsterdam, the Netherlands).

## 3 Results and discussion

### 3.1 Composition of pretreated tomato pomace

Hydroalcoholically extracted and dried TP, in which polar phenolic compounds had been reduced as described previously (Navajas Preciado et al., 2026b), exhibited a substantial dry matter content of 96% ± 1%. The matrix was rich in fibre, accounting for 92% ± 5% of the total composition. Within this matrix, two fibre fractions were identified: a major fraction consisting of 59% ± 7% soluble fibre and a minor fraction comprising 28% ± 2% insoluble fibre. Its compositional profile also showed low levels of fat (2.0% ± 0.5%) and protein (2.0% ± 0.7%), together with a moderate ash content (4.0% ± 0.4%). The carbohydrate fraction estimated as potentially accessible for fermentation (26% ± 4%) should therefore be interpreted as part of the fibre-rich carbohydrate pool, rather than as an additional centesimal component. This composition is particularly advantageous for organic-acid fermentation because ethanolic extraction minimises the disruption of bacterial homeostasis typically associated with polar phenolic compounds (Davidova et al., 2024). The potentially accessible carbohydrate fraction supports partial glucose replacement and the exploration of hydrolytic approaches, reinforcing the rationale for using TP as a circular feedstock (Hijosa-Valsero et al., 2019). The low protein content also indicates a nitrogen deficiency, supporting the evaluation of nitrogen supplementation during the optimisation strategy.

### 3.2 Sequential optimisation of organic acids production by *Lactiplantibacillus paraplantarum*

#### 3.2.1 Pre-screening

The pre-screening results shown in Figure 2 were used to focus on conditions that produced high biomass and stimulated acidification, an indicator of organic-acid accumulation by lactic acid bacteria. Conditions with high growth but insufficient acidification, or strong acidification but low growth, were excluded according to the criteria defined in Section 2.2.1. The Ref1 and Ref2 lines in Figure 2 are visual guides for acidification and growth, respectively. Three wells were selected: W8, W1 and W9. Each contained 0.5 mL of a 20% glucose solution in a final volume of 1.5 mL. Their initial pH ranged from 5.3 to 5.8, their final pH from 4.5 to 5.0, and their log_10_-transformed net increase in viable counts from 7.40 to 7.93. These wells were consistent with the typical response of *L. paraplantarum*, for which an initial pH of approximately 5.5–6.0 supports high final cell counts. As the medium approaches pH 5, the proportion of undissociated organic acids increases, which can inhibit growth and redirect metabolism towards cellular maintenance (Sun et al., 2024). Several regions near the intersection of the reference lines added little useful information for optimisation. Wells 17, 18, 4 and 15, for example, showed abrupt increases in pH before acidification (Supplementary Table S1), a pattern consistent with loss of viability and/or reduced acidifying activity under stress (Yin et al., 2025; Lu et al., 2026).

**FIGURE 2:**
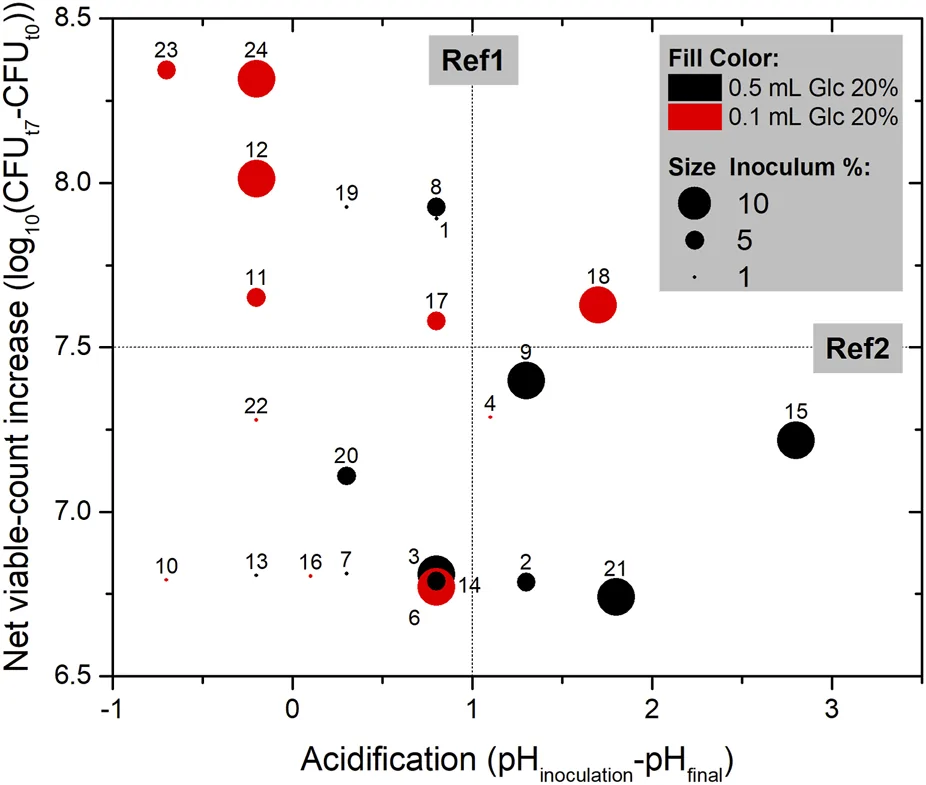
Pre-screening assessment of organic acid fermentation with *Lactiplantibacillus paraplantarum*. Numbers (1-24) refer to well positions; Ref1: reference line for interpreting the acidification criterion; Ref2: reference line for interpreting L. paraplantarum growth. The area near the intersection of the reference lines reflects the most suitable conditions according to the criteria defined in Section 2.2.1.

A central composite design (CCD) should cover a well-defined experimental region to improve statistical performance and exclude clearly unfavourable conditions (Szpisják-Gulyás et al., 2023). Accordingly, the selected region comprised an initial pH of 5.3–6.3, a 5% (vv^−1^) inoculum and 0.5 mL of 20% glucose (wv^−1^), equivalent to 81.9 g L^-1^ glucose. These settings allowed optimisation under carbon-saturating conditions similar to those reported previously (Ren et al., 2022) and provided fixed conditions for the subsequent fermentation model.

#### 3.2.2 RSM optimisation

With the experimental space defined by pre-screening (initial pH, carbon load and inoculum), a CCD was applied to maximise LA production from the mixed TP-glucose carbon source. This fix-then-optimise approach increased the statistical interpretability of the RSM by focusing the experiments on a domain with observable responses and reasonable kinetics. LA concentration (gL^-1^) was modelled as a function of the proportion of glucose replaced by TP (factor A, %), fermentation time (factor B, h) and initial pH (factor C; Supplementary Table S2). The general second-order polynomial is given in Equation 1. The fitted quadratic model adequately described the response (Figure 3), and the reduced model containing the significant terms was as follows:

**FIGURE 3:**
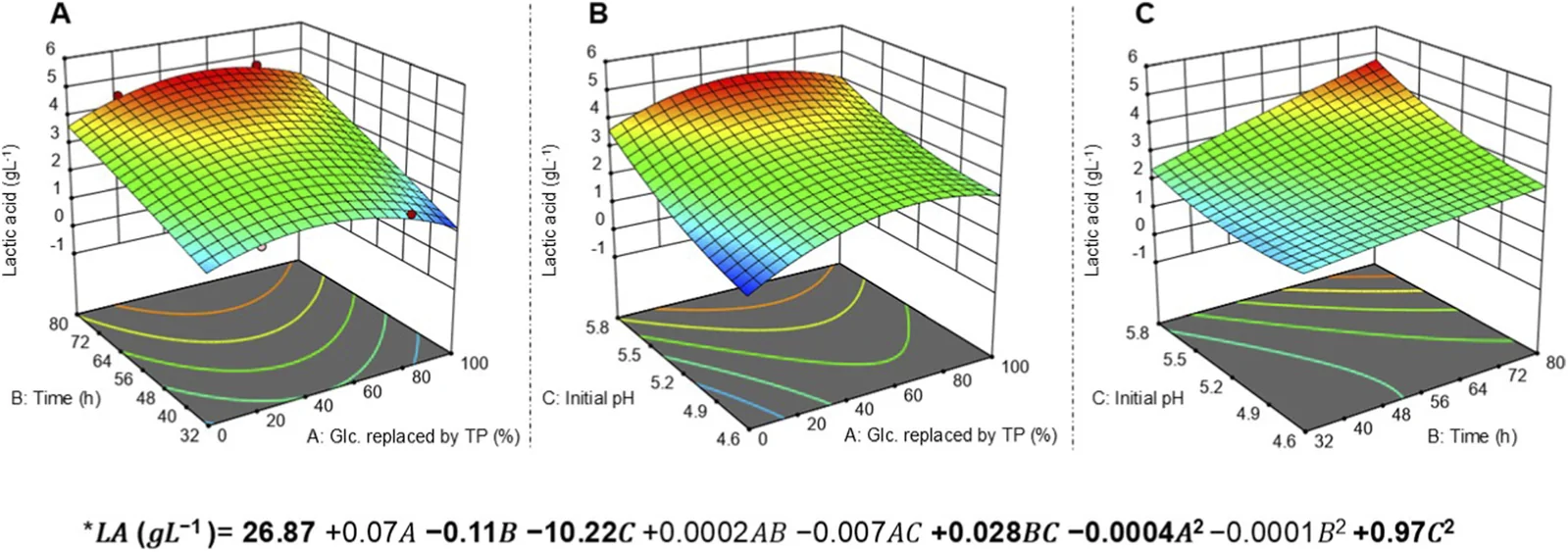
Optimisation of fermentative parameters of *Lactiplantibacillus paraplantarum*. Response surface methodology plots for lactic acid (LA) production represented as **(A)** factors A and B at the optimum value of C. **(B)** factors A and C at the optimum value of B; and **(C)** factors B and C at the optimum value of A. The equation uses actual factor values, with significant terms highlighted.

Reduced model containing the significant terms (Equation 2):

$$LA\;\left(gL^{-1}\right)=26.87-0.11B-10.22C+0.028BC-0.0004A^{2}+0.97C^{2}$$ (2)

ANOVA confirmed a robust fit (F = 30.91; p < 0.0001; R^2^ = 0.9755; adjusted R^2^ = 0.9439; predictive R^2^ = 0.7697) and a non-significant lack of fit (p > 0.05). Homoscedastic residuals, acceptable residual normality and low Cook’s distances supported the use of the model for prediction within the evaluated domain. From a mechanistic perspective, the terms retained in Equation 2 are consistent with fermentative physiology under minimal-medium conditions without active pH control. The effect of time (B) reflects diminishing returns compatible with product accumulation and acid stress. The initial-pH terms (C and C^2^) define an optimum at a moderate pH (approximately 5.5–6.5), where *L. paraplantarum* can remain active without severe acidification stress. The B × C interaction indicates that the effect of time depends on the initial pH, consistent with increasing weak-acid stress during fermentation. Previous studies likewise identify pH control or adjustment to intermediate values as important for maintaining growth and production and avoiding inhibitory conditions (Yankov, 2022; Bustos et al., 2025).

The response surfaces exhibited a plateau: increasing A and B raised LA only to approximately 5 g L^-1^, after which production stabilised. The constraint analysis likewise indicated that A > 70% and B > 80 h provided only marginal improvements. This behaviour is consistent with combined product inhibition, osmotic load and residual TP compounds, including organic acids and phenolic fractions not removed by hydroalcoholic extraction, which may impair microbial activity at high replacement levels (Yankov, 2022; Chabi et al., 2024; Deng et al., 2025).

A marked slowdown has been reported for *L. paraplantarum* in rich media above approximately 50–60 g L^-1^ LA, confirming the relevance of product inhibition (Lynch et al., 2026). In the present system, the apparent threshold was much lower, possibly because uncontrolled acidification acted together with the complex TP matrix.

Experimental verification supported the predictive performance of the model. The numerical optimum (A = 44.7%, B = 79.6 h and C = 5.77) produced 4.73 ± 0.26 g L^-1^ LA, compared with the predicted 4.68 g L^-1^ (deviation <2%; n = 3). The RSM therefore identified the region of maximum response within the evaluated domain. It also showed that, without nitrogen supplementation or pH control, inhibition rather than carbon availability constrained the system and guided the subsequent optimisation stages.

#### 3.2.3 Nitrogen-source optimisation

At the RSM optimum (pH 5.8, 79.6 h, 44.7% TP and 55.3% glucose), the reference without a nitrogen source produced 4.68 g L^-1^ LA. Treatments containing only inorganic nitrogen ((NH_4_)_2_SO_4_; S2, S5 and S8) produced 1.42–1.62 g L^-1^ LA. Thus, total nitrogen availability alone was not the limiting factor. Inorganic nitrogen could not meet the requirements of *L. paraplantarum* for amino acids, vitamins and anabolic precursors (Elmansy et al., 2023). This response is consistent with the marked auxotrophy of lactic acid bacteria, whose growth and fermentative metabolism depend on the supply of several amino acids and vitamins (Kim YH et al., 2024).

Conversely, organic nitrogen sources, particularly peptone and yeast extract, consistently improved fermentation performance, increasing LA to 5.12–7.68 g L^-1^ and raising the aGEC into quantified LA and AA to approximately 10% under the best-performing condition (Supplementary Table S3). Treatment S4 gave the highest response (7.68 ± 0.56 g L^-1^ LA and 0.59 ± 0.11 g L^-1^ AA; approximately 10% aGEC), followed by S1 (7.15 ± 0.44 g L^-1^ LA) and S7 (6.14 ± 0.31 g L^-1^ LA) (Figure 4A; Supplementary Table S3). This indicates that organic nitrogen increased not only LA accumulation but also the quantified acid signal relative to the theoretical glucose-equivalent input. Yeast extract supplies readily assimilable nitrogen compounds and cofactors, including B-group vitamins and nucleotide-rich fractions, which support biosynthesis and alleviate nutritional constraints (Pan-utai et al., 2022; Tao et al., 2023). The poorer response to formulations containing only inorganic nitrogen agrees with the requirements reported for lactic acid bacteria (Kim YH et al., 2024).

**FIGURE 4:**
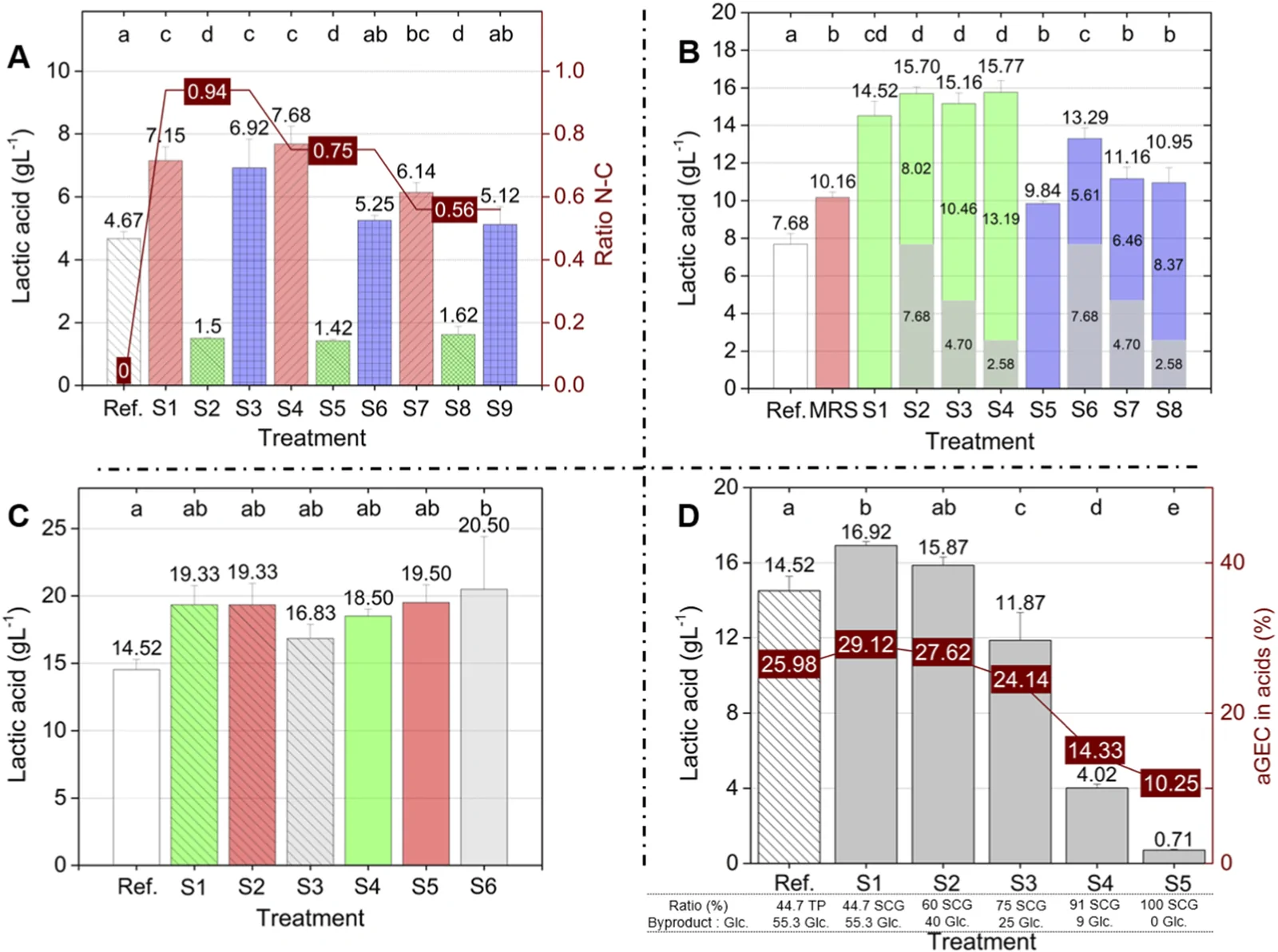
Sequential optimisation, combined process intensification and transferability of the basal fermentation conditions of *Lactiplantibacillus paraplantarum*. **(A)** Effects of nitrogen-source composition and the nitrogen-source:carbon-source mass ratio. White, RSM optimum; red, organic nitrogen only; green, inorganic nitrogen only; blue, mixed nitrogen sources. **(B)** Effects of initial pH and exogenous LA. White, nitrogen-optimisation reference; red, MRS reference; green, acetate buffer at pH 5.79; blue, acetate buffer at pH 4.5; grey, exogenous LA. **(C)** Combined intensification treatments (cellulase pretreatment and atmosphere). White, buffered reference; green, aerobic; red, anaerobic; grey, microaerobic. Hatched bars denote cellulase-pretreated samples. All treatments received 2.3 % CaCO_3_ (wv^-1^); because no basal condition containing CaCO_3_ alone was included, differences from the basal reference cannot be attributed separately to CaCO_3_, cellulase pretreatment or atmosphere. **(D)** Transfer of TP-derived basal conditions to SCG. The reference is the optimum buffered treatment; S1-S5 contain the SCG proportions shown beneath the panel. The apparent glucose-equivalent conversion (aGEC) was calculated as 100 × ([LA] + [AA])/81.88 gL^-1^. Because buffer-derived acetate was not subtracted, aGEC is a comparative, non-stoichiometric index rather than a closed carbon yield.For all figures data are presented as means ± standard deviation (SD) (n=3). Statistical significance between all groups (per figure) was evaluated via analysis of variance (ANOVA) followed by Tukey’s honestly significant difference (HSD) post-hoc test (p < 0.05), where distinct letters denote significant differences among treatments.

The greater biomass supported by organic nitrogen could also promote density-dependent regulation, including peptide-mediated quorum-sensing systems. Such regulation can increase cell death and complicate the monitoring of high-cell-density fermentations of lactic acid bacteria (Kuipers et al., 1998; Rizzello et al., 2014). Density-dependent responses may therefore have contributed to the subsequent decline in apparent carbohydrate conversion and LA productivity.

#### 3.2.4 pH buffering and lactic-acid supplementation

This experiment determined whether buffered pH control increased LA production and the aGEC into quantified organic acids, and whether accumulated lactate exerted additional inhibition. Once the best-performing nitrogen supplementation had been selected (Figure 4A; Supplementary Table S4), pH control had the greatest effect on intensification. At an initial pH of 5.8, the acetate-buffered medium produced 14.52 ± 0.76 g L^-1^ endogenous LA and 6.75 ± 0.46 g L^-1^ AA (Figure 4B; Supplementary Table S1), doubling the LA concentration observed under buffer-free conditions (7.68 g L^-1^) and exceeding that obtained at an initial pH of 4.5 (9.84 ± 0.13 g L^-1^; S5). The corresponding aGEC was approximately 26%, compared with approximately 10% after nitrogen optimisation. Because buffer-derived acetate was not subtracted, this is an apparent comparison rather than a closed conversion yield. This pattern confirms that, without pH-stat control, the progressive decrease in pH increases the undissociated acid fraction and amplifies weak-acid stress even when carbon remains available (Ojo and de Smidt, 2023; Pau et al., 2024). Exogenous LA reduced further endogenous LA production at both pH values. At pH 5.8, endogenous LA decreased from 14.52 g L^-1^ without added LA to 13.19, 10.46 and 8.02 g L^-1^ after addition of 2.58, 4.70 and 7.68 g L^-1^ LA, respectively. At pH 4.5, the corresponding decrease was more pronounced (9.84–8.37, 6.46 and 5.61 g L^-1^). Within the evaluated range, inhibition was therefore governed more strongly by pH-dependent acid stress than by total lactate concentration (Ojo and de Smidt, 2023; Sun et al., 2024).

### 3.3 Combined process intensification and product distribution

After defining the buffered basal window, LA production reached 14.52 ± 0.76 g L^-1^ under the nitrogen-supplemented baseline (Figure 4C, reference condition). This condition was then intensified by combining incubation atmosphere, ultrasound-cellulase pretreatment and CaCO_3_ supplementation. The final combined intensification treatment increased mean LA titres numerically to 16.83–20.50 g L^-1^, while AA concentrations among replicates ranged from 40.0 to 62.5 g L^-1^ and aGEC increased markedly. Because these factors were modified within the same experimental stage and no basal condition supplemented only with CaCO_3_ was included, their individual contributions cannot be separated. The highest mean LA titre was observed under microaerobic conditions without cellulase (20.50 g L^-1^), whereas cellulase-assisted treatments remained within a comparable range (16.83–19.33 g L^-1^). Neither cellulase pretreatment nor atmosphere significantly affected LA within the available comparisons. Thus, the principal outcome of the final stage was greater total acid accumulation dominated by acetate rather than selective intensification of LA production.

Figure 5 places this improvement in context by tracking the fermentation steps from basal optimisation to the final combined intensification treatment. During the early stages, aGEC was low and LA represented most of the quantified acid pool. As optimisation progressed, LA titres increased together with aGEC. However, the final combined treatment was accompanied by a marked rise in AA. At the highest aGEC values, LA represented only approximately 25%–30% of the quantified LA + AA pool; acetate therefore dominated the mixed-acid profile. Because aGEC is non-stoichiometric and includes buffer-derived acetate, this profile describes the distribution of quantified products rather than carbon flux. The final stage consequently prolonged the overall accumulation of acid without specifically enhancing lactic acid (LA) production. This alteration in product distribution indicates that selectivity, rather than fermentability alone, constitutes the primary metabolic limitation. It underscores the necessity for future strategies to redirect metabolism towards LA or another target platform acid.

**FIGURE 5:**
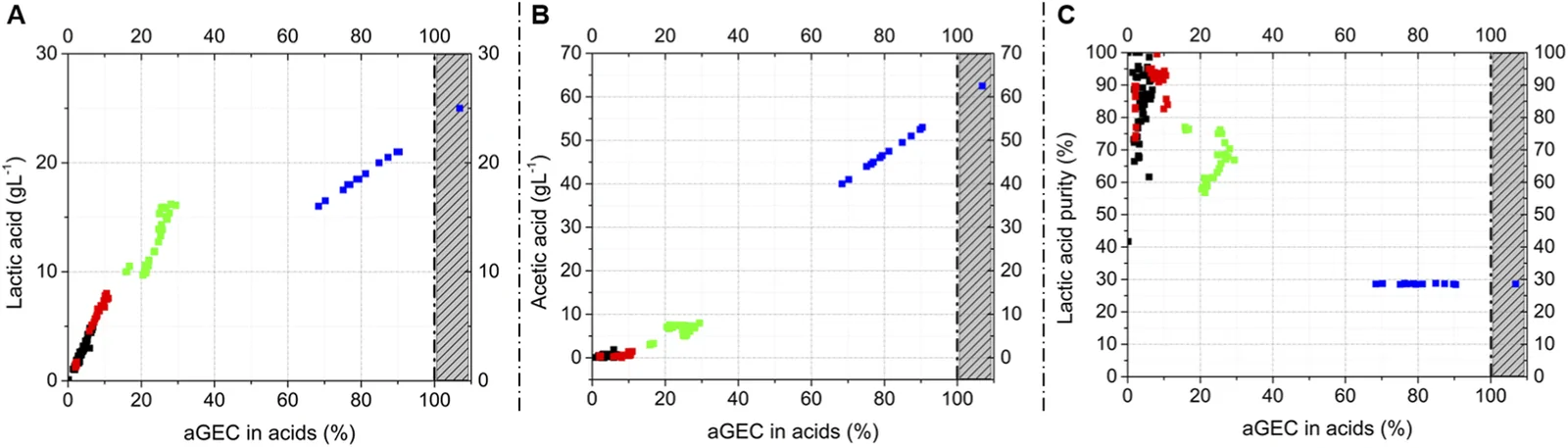
Sequential changes in lactic acid (LA), acetic acid (AA) and apparent glucose-equivalent conversion (aGEC) during basal optimisation and combined process intensification of *Lactiplantibacillus paraplantarum*. (A) LA concentration versus aGEC; (B) AA concentration versus aGEC; and (C) LA purity versus aGEC. Black, RSM; red, nitrogen-source optimisation; green, pH and exogenous-LA assessment; blue, combined intensification treatments. The aGEC was calculated as 100 × ([LA] + [AA])/81.88 g L‐1. Because buffer-derived acetate and other potentially fermentable carbon inputs were not fully represented in the theoretical denominator, the value above 100% is an artefact of this comparative, non-stoichiometric index and must not be interpreted as a closed carbon yield.

The final combined intensification treatment was associated with greater accumulation of the quantified acids, but the design cannot identify CaCO_3_, cellulase or atmosphere as the causal factor. Because CaCO_3_ was the only modification common to all treatments in this stage, additional buffering is a plausible mechanism: it may have prolonged pH stability and delayed acid inhibition (Hetényi et al., 2011; Shi et al., 2015; Balakrishnan et al., 2020). This interpretation requires a basal control containing CaCO_3_ without cellulase pretreatment or a modified atmosphere. From a process perspective, controlled-pH scale-up should isolate the buffering effect and assess the salt load and downstream-recovery burden associated with neutralisation, because conventional routes generate lactate salts and may produce environmentally problematic by-products such as lacto-gypsum (Marchesan et al., 2021; Ojo and de Smidt, 2023).

Overall, the sequential optimisation strategy identifies *L. paraplantarum* as a microbial platform for producing organic acids from media combining refined and residual carbon sources. However, because aGEC is an apparent index rather than a closed carbon balance, the fractions allocated to biomass, maintenance and unquantified metabolites cannot be resolved. The study therefore supports continued investigation of low-cost culture media based on renewable residual feedstocks rather than exclusively on refined substrates (Ojo and de Smidt, 2023; Kacaribu and Darwin, 2024). Nevertheless, important gaps remain before industrial deployment. Limited LA selectivity indicates that strain or process optimisation would be required to suppress competing pathways, including AA formation. Higher replacement of glucose by TP would also require more effective hydrolysate generation. Chemical hydrolysis has been proposed as a route for disrupting the lignocellulosic structure and producing fermentable streams containing 150–200 g L^-1^ glucose equivalents (Ren et al., 2022; Zhang et al., 2023). Its competitiveness with 1G industrial fermentation would, however, require dedicated scale-up and techno-economic assessment.

### 3.4 Transferability from TP to SCG

The optimised fermentation parameters from TP were applied to a second hydroalcoholically pretreated residue to evaluate their transferability to SCG, without conducting a full re-optimisation process. Figure 4D and Supplementary Table S6 show that these parameters remained effective only within a moderate SCG substitution range. At 44% and 60% replacement of standard glucose, LA reached 16.92 ± 0.20 and 15.87 ± 0.44 g L^-1^, respectively; AA reached 6.92 ± 0.08 and 6.78 ± 0.20 g L^-1^, and aGEC was approximately 28% and 29%. Above 60% replacement, both LA and aGEC declined, indicating a threshold beyond which substrate recalcitrance and residual inhibition overrode the transferred basal conditions (Yankov, 2022; Abolore et al., 2024). This behaviour is consistent with reports in which higher LA titres from SCG required sequential removal of inhibitory compounds and more effective hydrolysis, reaching approximately 22.8 g L^-1^ LA from SCG-derived streams (Kim S et al., 2024) and 48.6 g L^-1^ after optimised combined hydrolysis and fermentation (Wysocki et al., 2024). Transferability between residues is therefore feasible only within a defined substitution range in which carbon accessibility, residual inhibition and pH stability remain controlled.

### 3.5 q-^1^H NMR monitoring of organic acids

The complexity of this fermentation system highlights the need for direct monitoring tools that can support decision-making during fermentation. In this study, q-^1^H NMR was operationally applicable to monitoring LA and AA in complex 2G-biomass broths; the study did not constitute a full analytical validation.

q-^1^H NMR provided a rapid assessment of fermentation progress by enabling direct quantification of LA and AA in crude and clarified broth using DMSO as an internal standard. Under the specified conditions, the DMSO signal at 2.7 ppm remained distinct from the LA/lactate (1.30–1.35 ppm) and AA/acetate (1.95–2.05 ppm) resonances. The target signals in the aliphatic region were sufficiently resolved for direct integration (Supplementary Figures S1, S2), and minor pH-dependent shifts did not prevent quantification. Agreement between spectra from untreated broth and clarified supernatant supported the operational use of raw broth for routine monitoring without a clarification step. These observations are consistent with current q-^1^H NMR practice and its application to complex fermentation matrices (International Organization for Standardization, 2022; Gottstein et al., 2024; Forsberg et al., 2024), but they do not replace formal analytical validation.

The distribution of DMSO integrals (Figure 6) was widest during RSM and became more consistent in the later experimental groups, particularly during the pH/lactate and transferability stages. This trend suggests that sample preparation and integration improved as the workflow was standardised. Since acid concentrations were calculated relative to DMSO, the progressive stabilisation supports the operational repeatability of LA and AA monitoring. Nevertheless, it does not confirm analytical accuracy, precision, or robustness beyond the tested conditions (Roach et al., 2024).

**FIGURE 6:**
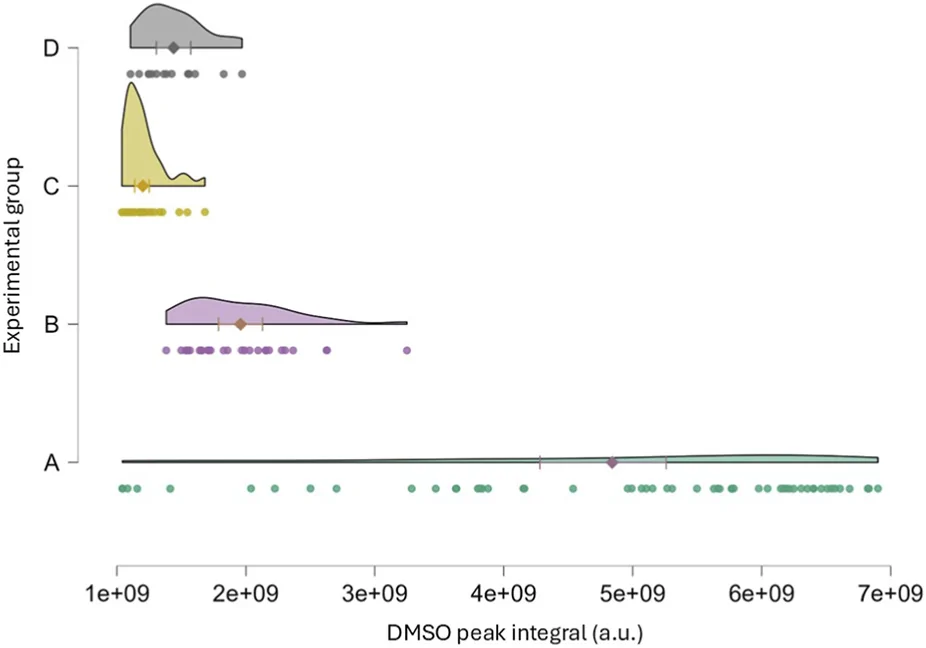
Distribution of the dimethyl sulfoxide (DMSO) peak integrals used to assess the operational repeatability of quantitative proton nuclear magnetic resonance (q-^1^H NMR) across the experimental groups analysed on the Bruker AvanceNeo 700 MHz. Green points represent the response surface methodology (RSM) stage; purple points represent nitrogen optimisation; yellow points represent the lactic acid and pH optimisation stage, and grey points represent the transferability assessment from tomato pomace (TP) to spent coffee grounds (SCG).

## 4 Conclusions

Under the laboratory conditions tested, hydroalcoholically pretreated TP and SCG could be fermented by *Lactiplantibacillus paraplantarum* as part of a cascade biorefinery. For TP, the sequential strategy identified an initial pH of 5.79, an operational nitrogen-source:carbon-source mass ratio of 0.75 and a 3:2 peptone-to-yeast-extract ratio as the basal conditions. Subsequent pH buffering increased LA production to 14.52 ± 0.76 g L^-1^. The final combined intensification treatment was associated with an aGEC of approximately 85%. However, this comparative, non-stoichiometric index includes buffer-derived acetate and is not a closed carbon yield. Because cellulase pretreatment, incubation atmosphere and CaCO_3_ supplementation were modified together, their individual contributions could not be resolved. The results support fermentation as a secondary valorisation stage after recovery of bioactive compounds from TP, while also showing that limited carbohydrate accessibility and product selectivity remain central constraints. More effective hydrolytic processes should therefore be evaluated to increase sugar accessibility and organic-acid production.

The TP-derived basal conditions remained useful for SCG only at 44%–60% glucose replacement, where LA reached 16.92–15.87 g L^-1^; higher replacement reduced both LA and aGEC. Thus, transferability does not imply that the residues behave identically, but that they can sustain fermentation within a defined range in which carbon accessibility, residual inhibition and pH stability remain controlled. At the highest aGEC values, acetate predominated, limiting process value when LA is the target platform chemical. Strain- and process-level optimisation should therefore seek to redirect metabolism towards LA. Industrial applicability will require controlled-pH scale-up, improved carbohydrate release, downstream-recovery studies and techno-economic assessment. The main contribution of this study is a sequential optimisation framework that distinguishes total-acid accumulation from LA selectivity and demonstrates the limited transferability of basal fermentation conditions between two residual feedstocks.

## Data Availability

The complete raw dataset supporting this study has been deposited in Zenodo under DOI 10.5281/zenodo.21273377.
