# Supplementary material for: Fermentative valorisation of agri-food residues: optimisation of Lactiplantibacillus paraplantarum fermentation conditions for the production of platform organic acids
Source: Front Bioeng Biotechnol. 2026 Sep 17;14:1935066. doi: 10.3389/fbioe.2026.1935066 (PMC13626635; doi:10.3389/fbioe.2026.1935066)
Supplement: Supplementary file 1 [file DataSheet1.docx]

Supplementary Material

**Figure S1.** Representative proton nuclear magnetic resonance spectra of lactic acid, glucose, dimethyl sulfoxide (DMSO) as internal standard, and tomato pomace fermentation broth.

**Figure S2.** Comparison of proton nuclear magnetic resonance spectra acquired from untreated fermentation broth and from the corresponding centrifuged and filtered supernatant.

| Run | C: Initial-Buffered pH | Lactic Acid exogenous | Lactic acid | | | |
| --- | --- | --- | --- | --- | --- | --- |
| BN-11-2025-S7-R2 | 4.5 | 4.7 | 11.06 | 6.36 |  |  |
| BN-11-2025-S7-R3 | 4.5 | 4.7 | 11.82 | 7.12 |  |  |
| BN-11-2025-S8-R1 | 4.5 | 2.58 | 10.55 | 7.97 | 8.37 | 0.79 |
| BN-11-2025-S8-R2 | 4.5 | 2.58 | 10.45 | 7.87 |  |  |
| BN-11-2025-S8-R3 | 4.5 | 2.58 | 11.87 | 9.29 |  |  |
| BN-11-2025-S9-R1 | - | - | 9.99 | 9.99 | 10.16 | 0.29 |
| BN-11-2025-S9-R2 | - | - | 9.99 | 9.99 |  |  |
| BN-11-2025-S9-R3 | - | - | 10.50 | 10.50 |  |  |

**Table S1.** Pre-screening dataset used to define the α-factor bounds for the basal fermentation parameters of Lactiplantibacillus paraplantarum. T_v_, total volume; CFU, Colony-forming units. Net CFU increase was calculated as log₁₀(CFUₜ₇ - CFUₜ₀).

|  | **Initial Conditions** | | | | **pH variations** | | | | | ***L. paraplantarum* growth** | | |
| --- | --- | --- | --- | --- | --- | --- | --- | --- | --- | --- | --- | --- |
| **Well Position** | **Inoculum (%, vv^-1^)** | **20% aqueous Glc. (mL)** | **T_v_ (mL)** | **Initial pH** | **Day 1** | **Day 2** | **Day 3** | **Day 7** | **Δ pH** | **CFUmL^-1^ (t0)** | **CFUmL^-1^ (t7)** | **Net CFU increase** |
| 1 | 1 | 0.5 | 1.5 | 5.3 | 5 | 5 | 5.2 | 4.5 | **-0.80** | 1.50E+05 | 7.80E+07 | **7.89** |
| 2 | 5 | 0.5 | 1.5 | 5.3 | 4.7 | 4.7 | 4.5 | 4 | **-1.30** | 4.00E+05 | 6.50E+06 | **6.79** |
| 3 | 10 | 0.5 | 1.5 | 5.3 | 4.7 | 4.2 | 3.8 | 4.5 | **-0.80** | 6.00E+04 | 6.50E+06 | **6.81** |
| 4 | 1 | 0.1 | 1.5 | 5.3 | 4.7 | 5.3 | 5.1 | 4.2 | **-1.10** | 1.10E+05 | 1.95E+07 | **7.29** |
| 5 | 5 | 0.1 | 1.5 | 5.3 | 4.7 | 5 | 4.7 | 4.5 | **-0.80** | 5.60E+05 | 6.50E+06 | **6.77** |
| 6 | 10 | 0.1 | 1.5 | 5.3 | 4.8 | 4.5 | 3.9 | 4.5 | **-0.80** | 6.00E+05 | 6.50E+06 | **6.77** |
| 7 | 1 | 0.5 | 1.5 | 5.8 | 6 | 5.7 | 5.8 | 5.5 | **-0.30** | 2.50E+04 | 6.50E+06 | **6.81** |
| 8 | 5 | 0.5 | 1.5 | 5.8 | 5.7 | 6.3 | 5.5 | 5 | **-0.80** | 5.00E+04 | 8.45E+07 | **7.93** |
| 9 | 10 | 0.5 | 1.5 | 5.8 | 5.5 | 5 | 4.5 | 4.5 | **-1.30** | 1.00E+06 | 2.60E+07 | **7.40** |
| 10 | 1 | 0.1 | 1.5 | 5.8 | 6.2 | 6 | 5.8 | 6.5 | **0.70** | 3.00E+05 | 6.50E+06 | **6.79** |
| 11 | 5 | 0.1 | 1.5 | 5.8 | 6 | 6 | 6 | 6 | **0.20** | 7.00E+05 | 4.55E+07 | **7.65** |
| 12 | 10 | 0.1 | 1.5 | 5.8 | 6 | 6.5 | 6.5 | 6 | **0.20** | 1.00E+06 | 1.04E+08 | **8.01** |
| 13 | 1 | 0.5 | 1.5 | 6.8 | 6.2 | 6.7 | 6.7 | 7 | **0.20** | 1.00E+05 | 6.50E+06 | **6.81** |
| 14 | 5 | 0.5 | 1.5 | 6.8 | 6.5 | 7.3 | 6 | 6 | **-0.80** | 3.50E+05 | 6.50E+06 | **6.79** |
| 15 | 10 | 0.5 | 1.5 | 6.8 | 6.5 | 7.5 | 5.1 | 4 | **-2.80** | 3.00E+06 | 1.95E+07 | **7.22** |
| 16 | 1 | 0.1 | 1.5 | 6.8 | 6.7 | 6.8 | 6.7 | 6.7 | **-0.10** | 1.40E+05 | 6.50E+06 | **6.80** |
| 17 | 5 | 0.1 | 1.5 | 6.8 | 6.5 | 7 | 6 | 6 | **-0.80** | 1.00E+06 | 3.90E+07 | **7.58** |
| 18 | 10 | 0.1 | 1.5 | 6.8 | 6 | 7.2 | 5.1 | 5.1 | **-1.70** | 3.00E+06 | 4.55E+07 | **7.63** |
| 19 | 1 | 0.5 | 1.5 | 6.3 | 6 | 8 | 6.5 | 6 | **-0.30** | 8.00E+04 | 8.45E+07 | **7.93** |
| 20 | 5 | 0.5 | 1.5 | 6.3 | 6 | 7.8 | 6 | 6 | **-0.30** | 1.50E+05 | 1.30E+07 | **7.11** |
| 21 | 10 | 0.5 | 1.5 | 6.3 | 6.2 | 6 | 5.5 | 4.5 | **-1.80** | 1.00E+06 | 6.50E+06 | **6.74** |
| 22 | 1 | 0.1 | 1.5 | 6.3 | 6.5 | 6.7 | 6.5 | 6.5 | **0.20** | 5.00E+05 | 1.95E+07 | **7.28** |
| 23 | 5 | 0.1 | 1.5 | 6.3 | 6.5 | 7.2 | 6.5 | 7 | **0.70** | 5.40E+05 | 2.21E+08 | **8.34** |
| 24 | 10 | 0.1 | 1.5 | 6.3 | 6 | 7.5 | 6.5 | 6.5 | **0.20** | 6.00E+05 | 2.08E+08 | **8.32** |

**Table S2.** Response surface methodology design matrix and lactic acid response for the optimisation of basal fermentation conditions in Lactiplantibacillus paraplantarum. TP, tomato pomace; F_v_, final volume; LA, lactic acid; AA, acetic acid.

|  | **Factor A** | **Mixed in 30 mL (F_v_)** | | | **Factor C** | **Factor B** | **Response** | |
| --- | --- | --- | --- | --- | --- | --- | --- | --- |
| **Run** | **Glc. replace by TP (%)** |  | **TP (g)** | **Glc. (g)** | **C: Initial pH** | **B:Time (h)** | **LA (gL^-1^)** | **AA (gL^-1^)** |
| 1 | 20.27 |  | 1.42 | 1.59 | 4.60 | 32.00 | 1.72 | 0.14 |
| 1 | 20.27 |  | 1.42 | 1.59 | 4.60 | 32.00 | 1.67 | 0.2 |
| 1 | 20.27 |  | 1.42 | 1.59 | 4.60 | 32.00 | 1.32 | 0.17 |
| 2 | 79.73 |  | 5.58 | 0.41 | 5.80 | 80.00 | 4.92 | 0.64 |
| 2 | 79.73 |  | 5.58 | 0.41 | 5.80 | 80.00 | 4.67 | 0.71 |
| 2 | 79.73 |  | 5.58 | 0.41 | 5.80 | 80.00 | 4.41 | 0.74 |
| 3 | 20.27 |  | 1.42 | 1.59 | 4.60 | 80.00 | 3.20 | 0.24 |
| 3 | 20.27 |  | 1.42 | 1.59 | 4.60 | 80.00 | 3.14 | 0.24 |
| 3 | 20.27 |  | 1.42 | 1.59 | 4.60 | 80.00 | 3.65 | 0.27 |
| 4 | 50.00 |  | 3.50 | 1.00 | 6.21 | 56.00 | 4.56 | 0.44 |
| 4 | 50.00 |  | 3.50 | 1.00 | 6.21 | 56.00 | 4.51 | 0.44 |
| 4 | 50.00 |  | 3.50 | 1.00 | 6.21 | 56.00 | 4.82 | 0.44 |
| 5 | 50.00 |  | 3.50 | 1.00 | 5.20 | 56.00 | 2.64 | 0.44 |
| 5 | 50.00 |  | 3.50 | 1.00 | 5.20 | 56.00 | 2.69 | 0.54 |
| 5 | 50.00 |  | 3.50 | 1.00 | 5.20 | 56.00 | 2.74 | 0.41 |
| 6 | 50.00 |  | 3.50 | 1.00 | 5.20 | 96.36 | 3.70 | 0.54 |
| 6 | 50.00 |  | 3.50 | 1.00 | 5.20 | 96.36 | 3.75 | 0.57 |
| 6 | 50.00 |  | 3.50 | 1.00 | 5.20 | 96.36 | 3.55 | 0.54 |
| 7 | 50.00 |  | 3.50 | 1.00 | 5.20 | 56.00 | 2.33 | 0.41 |
| 7 | 50.00 |  | 3.50 | 1.00 | 5.20 | 56.00 | 2.28 | - |
| 7 | 50.00 |  | 3.50 | 1.00 | 5.20 | 56.00 | 2.33 | 0.44 |
| 8 | 79.73 |  | 5.58 | 0.41 | 5.80 | 32.00 | 1.88 | 0.74 |
| 8 | 79.73 |  | 5.58 | 0.41 | 5.80 | 32.00 | 1.88 | 0.51 |
| 8 | 79.73 |  | 5.58 | 0.41 | 5.80 | 32.00 | 2.38 | 0.64 |
| 9 | 50.00 |  | 3.50 | 1.00 | 5.20 | 15.64 | 1.01 | 0.37 |
| 9 | 50.00 |  | 3.50 | 1.00 | 5.20 | 15.64 | 1.01 | 0.51 |
| 9 | 50.00 |  | 3.50 | 1.00 | 5.20 | 15.64 | 1.07 | 0.41 |
| 10 | 79.73 |  | 5.58 | 0.41 | 4.60 | 32.00 | 1.88 | - |
| 10 | 79.73 |  | 5.58 | 0.41 | 4.60 | 32.00 | 1.62 | 0.57 |
| 10 | 79.73 |  | 5.58 | 0.41 | 4.60 | 32.00 | 1.78 | 0.54 |
| 11 | 0.00 |  | 0.00 | 2.00 | 5.20 | 56.00 | 0.05 | 0.07 |
| 11 | 0.00 |  | 0.00 | 2.00 | 5.20 | 56.00 | 0.10 | - |
| 11 | 0.00 |  | 0.00 | 2.00 | 5.20 | 56.00 | 0.05 | - |
| 12 | 20.27 |  | 1.42 | 1.59 | 5.80 | 80.00 | 4.82 | 0.07 |
| 12 | 20.27 |  | 1.42 | 1.59 | 5.80 | 80.00 | 4.26 | 0.2 |
| 12 | 20.27 |  | 1.42 | 1.59 | 5.80 | 80.00 | 4.16 | 0.27 |
| 13 | 50.00 |  | 3.50 | 1.00 | 5.20 | 56.00 | 2.99 | 1.86 |
| 13 | 50.00 |  | 3.50 | 1.00 | 5.20 | 56.00 | 2.64 | - |
| 13 | 50.00 |  | 3.50 | 1.00 | 5.20 | 56.00 | 2.69 | 0.54 |
| 14 | 50.00 |  | 3.50 | 1.00 | 4.19 | 56.00 | 2.33 | 0.41 |
| 14 | 50.00 |  | 3.50 | 1.00 | 4.19 | 56.00 | 2.43 | 0.44 |
| 14 | 50.00 |  | 3.50 | 1.00 | 4.19 | 56.00 | 2.49 | 0.47 |
| 15 | 100.00 |  | 7.00 | 0.00 | 5.20 | 56.00 | 1.78 | 0.85 |
| 15 | 100.00 |  | 7.00 | 0.00 | 5.20 | 56.00 | 1.62 | 0.78 |
| 15 | 100.00 |  | 7.00 | 0.00 | 5.20 | 56.00 | 1.67 | 0.78 |
| 16 | 79.73 |  | 5.58 | 0.41 | 4.60 | 80.00 | 3.30 | 0.85 |
| 16 | 79.73 |  | 5.58 | 0.41 | 4.60 | 80.00 | 2.89 | 0.68 |
| 16 | 79.73 |  | 5.58 | 0.41 | 4.60 | 80.00 | 2.74 | 0.64 |
| 17 | 50.00 |  | 3.50 | 1.00 | 5.20 | 56.00 | 2.54 | 0.14 |
| 17 | 50.00 |  | 3.50 | 1.00 | 5.20 | 56.00 | 2.38 | 0.37 |
| 17 | 50.00 |  | 3.50 | 1.00 | 5.20 | 56.00 | 2.79 | 0.61 |
| 18 | 20.27 |  | 1.42 | 1.59 | 5.80 | 32.00 | 1.07 | 0.07 |
| 18 | 20.27 |  | 1.42 | 1.59 | 5.80 | 32.00 | 2.28 | 0.1 |
| 18 | 20.27 |  | 1.42 | 1.59 | 5.80 | 32.00 | 2.03 | 0.17 |
| 19 | 50.00 |  | 3.50 | 1.00 | 5.20 | 56.00 | 2.69 | 0.41 |
| 19 | 50.00 |  | 3.50 | 1.00 | 5.20 | 56.00 | 3.04 | 0.37 |
| 19 | 50.00 |  | 3.50 | 1.00 | 5.20 | 56.00 | 3.20 | 0.61 |
| Optimum | 44.50 |  | 1.57 | 1.11 | 5.80 | 79.60 | 4.51 | 0.34 |
| Optimum | 44.50 |  | 1.57 | 1.11 | 5.80 | 79.60 | 4.82 | 0.3 |

**Table S3.** Experimental matrix for nitrogen-source optimisation, showing the nitrogen-source:carbon-source mass ratio (NS:CS) and the organic/inorganic nitrogen composition used to maximise organic acid production by Lactiplantibacillus paraplantarum. TP; tomato pomace; nitrogen-to-carbon (N:C) ratio; LA, lactic acid; AA, acetic acid.

| **Sample** | **TP (g)** | **Glc. (g)** | **C: Initial pH** | **B: Time (h)** | **Peptone (g)** | **Yeast Extract (g)** | **NH_4_SO_2_ (g)** | **NS:CS mass ratio** | **LA (gL^-1^)** | **AA (gL^-1^)** |
| --- | --- | --- | --- | --- | --- | --- | --- | --- | --- | --- |
| S1 | 1.56 | 1.11 | 5.8 | 79.58 | 1.5 | 1 | 0 | 0.94 | 7.46 | 1.25 |
| S1 | 1.56 | 1.11 | 5.8 | 79.58 | 1.5 | 1 | 0 | 0.94 | 6.75 | 1.42 |
| S1 | 1.56 | 1.11 | 5.8 | 79.58 | 1.5 | 1 | 0 | 0.94 | 7.56 | 1.45 |
| S2 | 1.56 | 1.11 | 5.8 | 79.58 | 0 | 0 | 2.5 | 0.94 | 1.47 | 0.44 |
| S2 | 1.56 | 1.11 | 5.8 | 79.58 | 0 | 0 | 2.5 | 0.94 | 1.52 | 0.24 |
| S2 | 1.56 | 1.11 | 5.8 | 79.58 | 0 | 0 | 2.5 | 0.94 | 1.47 | 0.17 |
| S3 | 1.56 | 1.11 | 5.8 | 79.58 | 0.75 | 0.5 | 1.25 | 0.94 | 6.59 | 0.03 |
| S3 | 1.56 | 1.11 | 5.8 | 79.58 | 0.75 | 0.5 | 1.25 | 0.94 | 7.81 | 0.47 |
| S3 | 1.56 | 1.11 | 5.8 | 79.58 | 0.75 | 0.5 | 1.25 | 0.94 | 6.04 | 0.47 |
| S4 | 1.56 | 1.11 | 5.8 | 79.58 | 1.2 | 0.8 | 0 | 0.75 | 6.90 | 0.47 |
| S4 | 1.56 | 1.11 | 5.8 | 79.58 | 1.2 | 0.8 | 0 | 0.75 | 8.01 | 0.61 |
| S4 | 1.56 | 1.11 | 5.8 | 79.58 | 1.2 | 0.8 | 0 | 0.75 | 7.35 | 0.68 |
| S5 | 1.56 | 1.11 | 5.8 | 79.58 | 0 | 0 | 2 | 0.75 | 1.42 | 0.3 |
| S5 | 1.56 | 1.11 | 5.8 | 79.58 | 0 | 0 | 2 | 0.75 | 1.47 | 0.3 |
| S5 | 1.56 | 1.11 | 5.8 | 79.58 | 0 | 0 | 2 | 0.75 | 1.37 | 0.47 |
| S6 | 1.56 | 1.11 | 5.8 | 79.58 | 0.6 | 0.4 | 1 | 0.75 | 5.07 | 0.27 |
| S6 | 1.56 | 1.11 | 5.8 | 79.58 | 0.6 | 0.4 | 1 | 0.75 | 5.38 | 0.47 |
| S6 | 1.56 | 1.11 | 5.8 | 79.58 | 0.6 | 0.4 | 1 | 0.75 | 5.12 | 0.27 |
| S7 | 1.56 | 1.11 | 5.8 | 79.58 | 0.9 | 0.6 | 0 | 0.56 | 6.44 | 0.54 |
| S7 | 1.56 | 1.11 | 5.8 | 79.58 | 0.9 | 0.6 | 0 | 0.56 | 6.39 | 0.64 |
| S7 | 1.56 | 1.11 | 5.8 | 79.58 | 0.9 | 0.6 | 0 | 0.56 | 5.88 | 0.51 |
| S8 | 1.56 | 1.11 | 5.8 | 79.58 | 0 | 0 | 1.5 | 0.56 | 1.22 | 0.44 |
| S8 | 1.56 | 1.11 | 5.8 | 79.58 | 0 | 0 | 1.5 | 0.56 | 1.52 | 0.2 |
| S8 | 1.56 | 1.11 | 5.8 | 79.58 | 0 | 0 | 1.5 | 0.56 | 1.72 | 0.2 |
| S9 | 1.56 | 1.11 | 5.8 | 79.58 | 0.45 | 0.3 | 0.75 | 0.56 | 4.97 | 0.34 |
| S9 | 1.56 | 1.11 | 5.8 | 79.58 | 0.45 | 0.3 | 0.75 | 0.56 | 4.56 | 0.24 |
| S9 | 1.56 | 1.11 | 5.8 | 79.58 | 0.45 | 0.3 | 0.75 | 0.56 | 5.68 | 0.37 |

**Table S4.** Effect of buffered pH and exogenous lactic acid on basal fermentation performance and endogenous lactic acid production by Lactiplantibacillus paraplantarum. TP; tomato pomace; F_v_, final volume; LA, lactic acid; AA, acetic acid.

| **Sample** | **Peptone (g)** | **Yeast Extract (g)** | **TP (g)** | **Glc. (g)** | **Time (h)** | **Fv (mL)** | **C: Initial-Buffered pH (u)** | **LA exogenous (gL^-1^)** | **Total LA (gL^-1^)** | **Endogenous LA (gL^-1^)** | **AA (gL^-1^)** |
| --- | --- | --- | --- | --- | --- | --- | --- | --- | --- | --- | --- |
| S1 | 1.2 | 0.8 | 1.56 | 1.11 | 79.58 | 30 | 5.8 | 0 | 14.30 | 14.30 | 6.59 |
| S1 | 1.2 | 0.8 | 1.56 | 1.11 | 79.58 | 30 | 5.8 | 0 | 13.90 | 13.90 | 6.39 |
| S1 | 1.2 | 0.8 | 1.56 | 1.11 | 79.58 | 30 | 5.8 | 0 | 15.37 | 15.37 | 7.27 |
| S2 | 1.2 | 0.8 | 1.56 | 1.11 | 79.58 | 30 | 5.8 | 7.68 | 15.92 | 8.24 | 5.31 |
| S2 | 1.2 | 0.8 | 1.56 | 1.11 | 79.58 | 30 | 5.8 | 7.68 | 15.32 | 7.64 | 5.11 |
| S2 | 1.2 | 0.8 | 1.56 | 1.11 | 79.58 | 30 | 5.8 | 7.68 | 15.87 | 8.19 | 4.97 |
| S3 | 1.2 | 0.8 | 1.56 | 1.11 | 79.58 | 30 | 5.8 | 4.7 | 14.81 | 10.11 | 7.27 |
| S3 | 1.2 | 0.8 | 1.56 | 1.11 | 79.58 | 30 | 5.8 | 4.7 | 15.82 | 11.12 | 6.09 |
| S3 | 1.2 | 0.8 | 1.56 | 1.11 | 79.58 | 30 | 5.8 | 4.7 | 14.86 | 10.16 | 7.3 |
| S4 | 1.2 | 0.8 | 1.56 | 1.11 | 79.58 | 30 | 5.8 | 2.58 | 16.18 | 13.60 | 6.83 |
| S4 | 1.2 | 0.8 | 1.56 | 1.11 | 79.58 | 30 | 5.8 | 2.58 | 15.06 | 12.48 | 6.9 |
| S4 | 1.2 | 0.8 | 1.56 | 1.11 | 79.58 | 30 | 5.8 | 2.58 | 16.08 | 13.50 | 7.98 |
| S5 | 1.2 | 0.8 | 1.56 | 1.11 | 79.58 | 30 | 4.5 | 0 | 9.69 | 9.69 | 7.07 |
| S5 | 1.2 | 0.8 | 1.56 | 1.11 | 79.58 | 30 | 4.5 | 0 | 9.94 | 9.94 | 7.07 |
| S5 | 1.2 | 0.8 | 1.56 | 1.11 | 79.58 | 30 | 4.5 | 0 | 9.89 | 9.89 | 7.54 |
| S6 | 1.2 | 0.8 | 1.56 | 1.11 | 79.58 | 30 | 4.5 | 7.68 | 12.73 | 5.05 | 7.47 |
| S6 | 1.2 | 0.8 | 1.56 | 1.11 | 79.58 | 30 | 4.5 | 7.68 | 13.29 | 5.61 | 7.4 |
| S6 | 1.2 | 0.8 | 1.56 | 1.11 | 79.58 | 30 | 4.5 | 7.68 | 13.85 | 6.17 | 7.2 |
| S7 | 1.2 | 0.8 | 1.56 | 1.11 | 79.58 | 30 | 4.5 | 4.7 | 10.60 | 5.90 | 6.69 |
| S7 | 1.2 | 0.8 | 1.56 | 1.11 | 79.58 | 30 | 4.5 | 4.7 | 11.06 | 6.36 | 7 |
| S7 | 1.2 | 0.8 | 1.56 | 1.11 | 79.58 | 30 | 4.5 | 4.7 | 11.82 | 7.12 | 7.4 |
| S8 | 1.2 | 0.8 | 1.56 | 1.11 | 79.58 | 30 | 4.5 | 2.58 | 10.55 | 7.97 | 7.4 |
| S8 | 1.2 | 0.8 | 1.56 | 1.11 | 79.58 | 30 | 4.5 | 2.58 | 10.45 | 7.87 | 7.17 |
| S8 | 1.2 | 0.8 | 1.56 | 1.11 | 79.58 | 30 | 4.5 | 2.58 | 11.87 | 9.29 | 7.47 |
| S9 | MRS | | | | 79.58 | 30 | - | - | 9.99 | 9.99 | - |
| S9 | MRS | | | | 79.58 | 30 | - | - | 9.99 | 9.99 | - |
| S9 | MRS | | | | 79.58 | 30 | - | - | 10.50 | 10.50 | - |

| **Sample** | **Pep (g)** | **YE (g)** | **TP (g)** | **Glc. (g)** | **Time (h)** | **Fv (mL)** | **Initial-Buffered pH (u)** | **0.02 g Cell. /Sample** | **Atmosphere** | **LA (gL^-1^)** | **AA (gL^-1^)** | **LA+AA (gL^-1^)** | **aGEC, comparative index (%)** |
| --- | --- | --- | --- | --- | --- | --- | --- | --- | --- | --- | --- | --- | --- |
| **1** | 1.2 | 0.8 | 1.56 | 1.11 | 79.58 | 30 | 5.8 | YES | Aerobic | 21.00 | 53 | 74.00 | 90.38 |
| **2** | 1.2 | 0.8 | 1.56 | 1.11 | 79.58 | 30 | 5.8 | YES | Aerobic | 18.50 | 46 | 64.50 | 78.77 |
| **3** | 1.2 | 0.8 | 1.56 | 1.11 | 79.58 | 30 | 5.8 | YES | Aerobic | 18.50 | 46.5 | 65.00 | 79.38 |
| **4** | 1.2 | 0.8 | 1.56 | 1.11 | 79.58 | 30 | 5.8 | YES | Anaerobic | 17.50 | 44 | 61.50 | 75.11 |
| **5** | 1.2 | 0.8 | 1.56 | 1.11 | 79.58 | 30 | 5.8 | YES | Anaerobic | 20.50 | 51 | 71.50 | 87.32 |
| **6** | 1.2 | 0.8 | 1.56 | 1.11 | 79.58 | 30 | 5.8 | YES | Anaerobic | 20.00 | 49.5 | 69.50 | 84.88 |
| **7** | 1.2 | 0.8 | 1.56 | 1.11 | 79.58 | 30 | 5.8 | YES | Microaerobic | 16.50 | 41 | 57.50 | 70.22 |
| **8** | 1.2 | 0.8 | 1.56 | 1.11 | 79.58 | 30 | 5.8 | YES | Microaerobic | 18.00 | 45 | 63.00 | 76.94 |
| **9** | 1.2 | 0.8 | 1.56 | 1.11 | 79.58 | 30 | 5.8 | YES | Microaerobic | 16.00 | 40 | 56.00 | 68.39 |
| **10** | 1.2 | 0.8 | 1.56 | 1.11 | 79.58 | 30 | 5.8 | NO | Aerobic | 19.00 | 47.5 | 66.50 | 81.22 |
| **11** | 1.2 | 0.8 | 1.56 | 1.11 | 79.58 | 30 | 5.8 | NO | Aerobic | 18.00 | 45 | 63.00 | 76.94 |
| **12** | 1.2 | 0.8 | 1.56 | 1.11 | 79.58 | 30 | 5.8 | NO | Aerobic | 18.50 | 46.5 | 65.00 | 79.38 |
| **13** | 1.2 | 0.8 | 1.56 | 1.11 | 79.58 | 30 | 5.8 | NO | Anaerobic | 18.50 | 46.5 | 65.00 | 79.38 |
| **14** | 1.2 | 0.8 | 1.56 | 1.11 | 79.58 | 30 | 5.8 | NO | Anaerobic | 21.00 | 52.5 | 73.50 | 89.77 |
| **15** | 1.2 | 0.8 | 1.56 | 1.11 | 79.58 | 30 | 5.8 | NO | Anaerobic | 19.00 | 47.5 | 66.50 | 81.22 |
| **16** | 1.2 | 0.8 | 1.56 | 1.11 | 79.58 | 30 | 5.8 | NO | Microaerobic | 18.00 | 44.5 | 62.50 | 76.33 |
| **17** | 1.2 | 0.8 | 1.56 | 1.11 | 79.58 | 30 | 5.8 | NO | Microaerobic | 18.50 | 46 | 64.50 | 78.77 |
| **18** | 1.2 | 0.8 | 1.56 | 1.11 | 79.58 | 30 | 5.8 | NO | Microaerobic | 25.00 | 62.5 | 87.50 | 106.86 |

**Table S5.** Experimental matrix for synergistic process intensification, including ultrasound-cellulase pretreatment and incubation atmosphere, during organic acid fermentation Lactiplantibacillus paraplantarum. Pep, peptone; YE, yeast extract; TP; tomato pomace; Fv, final volume; Cell., cellulase; LA, lactic acid; AA, acetic acid; Glc. Eq., glucose equivalents. Apparent Glc. Eq. conversion (aGEC) into quantified acids was used as a comparative index and does not represent a closed carbon balance. All treatments listed in Table S5 received 2.3 % wv^-1^ CaCO₃. No basal + CaCO₃ control was included; therefore, differences from the basal reference cannot be attributed separately to CaCO₃, cellulase pretreatment or atmosphere. aGEC (%) = 100 × ([LA] + [AA])/81.88 gL^-1^, where the denominator is the theoretical glucose-equivalent concentration. Buffer-derived acetate was not subtracted and other carbon inputs and outputs were not included; values, including the observation above 100 %, are comparative artefacts of the index rather than closed yields.

**Table S6**. Transferability assessment of the optimised basal fermentation conditions from tomato pomace to spent coffee grounds in Lactiplantibacillus paraplantarum fermentation. SCG, spent coffee grounds; F_v_, final volume; LA, lactic acid; AA, acetic acid.

| **Sample** | **Glc. replaced by SCG (%)** | **Glc. (%)** | **Peptone (g)** | **Yeast Extract (g)** | **Time (h)** | **Fv (mL)** | **Initial-Buffered pH (u)** | **LA (gL^-1^)** | **AA (gL^-1^)** |
| --- | --- | --- | --- | --- | --- | --- | --- | --- | --- |
| S1 | 44 | 56 | 1.2 | 0.8 | 79.58 | 30 | 5.8 | 16.99 | 6.83 |
| S1 | 44 | 56 | 1.2 | 0.8 | 79.58 | 30 | 5.8 | 17.09 | 6.96 |
| S1 | 44 | 56 | 1.2 | 0.8 | 79.58 | 30 | 5.8 | 16.69 | 6.96 |
| S2 | 60 | 40 | 1.2 | 0.8 | 79.58 | 30 | 5.8 | 15.37 | 6.56 |
| S2 | 60 | 40 | 1.2 | 0.8 | 79.58 | 30 | 5.8 | 16.08 | 6.96 |
| S2 | 60 | 40 | 1.2 | 0.8 | 79.58 | 30 | 5.8 | 16.18 | 6.83 |
| S3 | 75 | 25 | 1.2 | 0.8 | 79.58 | 30 | 5.8 | 13.54 | 8.99 |
| S3 | 75 | 25 | 1.2 | 0.8 | 79.58 | 30 | 5.8 | 10.75 | 7.2 |
| S3 | 75 | 25 | 1.2 | 0.8 | 79.58 | 30 | 5.8 | 11.31 | 7.51 |
| S4 | 91 | 9 | 1.2 | 0.8 | 79.58 | 30 | 5.8 | 4.16 | 7.74 |
| S4 | 91 | 9 | 1.2 | 0.8 | 79.58 | 30 | 5.8 | 4.11 | 7.78 |
| S4 | 91 | 9 | 1.2 | 0.8 | 79.58 | 30 | 5.8 | 3.80 | 7.61 |
| S5 | 100 | 0 | 1.2 | 0.8 | 79.58 | 30 | 5.8 | 0.66 | 7.95 |
| S5 | 100 | 0 | 1.2 | 0.8 | 79.58 | 30 | 5.8 | 0.71 | 7.64 |
| S5 | 100 | 0 | 1.2 | 0.8 | 79.58 | 30 | 5.8 | 0.76 | 7.47 |
